# Supplementary material for: Machine Learning Accelerates Crystallization for Structure Determination
Source: Angew Chem Int Ed Engl. 2026 May 4;65(25):e1218503. doi: 10.1002/anie.1218503 (PMC13266958; doi:10.1002/anie.1218503)

# checkCIF/PLATON report

Structure factors have been supplied for datablock(s) lcz032

THIS REPORT IS FOR GUIDANCE ONLY. IF USED AS PART OF A REVIEW PROCEDURE FOR PUBLICATION, IT SHOULD NOT REPLACE THE EXPERTISE OF AN EXPERIENCED CRYSTALLOGRAPHIC REFEREE.

No syntax errors found. CIF dictionary Interpreting this report

# Datablock: lcz032

---

| | | | |
|---|---|---|---|
| Bond precision: | C-C = 0.0114 A | | Wavelength=1.54184 |
| Cell: | a=8.7328(2) | b=18.1114(3) | c=22.8152(3) |
| | alpha=90 | beta=100.574(2) | gamma=90 |
| Temperature: | 100 K | | |

| | Calculated | Reported |
|---|---|---|
| Volume | 3547.25(11) | 3547.25(11) |
| Space group | P 21/c | P 1 21/c 1 |
| Hall group | -P 2ybc | -P 2ybc |
| Moiety formula | C27 H17 Ag3 F18 N6 O4 | C15 H3 Ag3 F18 N6, C12 H14 O4 |
| Sum formula | C27 H17 Ag3 F18 N6 O4 | C27 H15 Ag3 F18 N6 O4 |
| Mr | 1155.08 | 1153.06 |
| Dx,g cm-3 | 2.163 | 2.159 |
| Z | 4 | 4 |
| Mu (mm-1) | 14.463 | 14.462 |
| F000 | 2224.0 | 2216.0 |
| F000’ | 2234.73 | |
| h,k,lmax | 10,22,28 | 10,22,28 |
| Nref | 7282 | 6985 |
| Tmin,Tmax | 0.078,0.132 | 0.486,1.000 |
| Tmin’ | 0.012 | |

Correction method= # Reported T Limits: Tmin=0.486 Tmax=1.000
AbsCorr = MULTI-SCAN

```
Data completeness= 0.959            Theta(max)= 74.690

R(reflections)= 0.0530( 6120)            wR2(reflections)=
                                          0.1506( 6985)
S = 1.035                Npar= 534
```

---

```
The following ALERTS were generated. Each ALERT has the format
       test-name_ALERT_alert-type_alert-level.
Click on the hyperlinks for more details of the test.
```

---

## Alert level C

```
PLAT041_ALERT_1_C Calc. and Reported SumFormula    Strings  Differ     Please Check
              Calc: C27 H17 Ag3 F18 N6 O4
              Rep.: C27 H15 Ag3 F18 N6 O4
PLAT042_ALERT_1_C Calc. and Reported MoietyFormula Strings  Differ     Please Check
              Calc: C27 H17 Ag3 F18 N6 O4
              Rep.: C15 H3 Ag3 F18 N6, C12 H14 O4
PLAT043_ALERT_1_C Calculated and Reported Mol. Weight Differ by ..       2.02 Check
PLAT068_ALERT_1_C Reported F000 Differs from Calcd (or Missing)...     Please Check
PLAT094_ALERT_2_C Ratio of Maximum / Minimum Residual Density ....       2.07 Report
PLAT213_ALERT_2_C Atom F010            has ADP max/min Ratio .....        3.2 prolat
PLAT213_ALERT_2_C Atom F015            has ADP max/min Ratio .....        3.3 prolat
PLAT213_ALERT_2_C Atom F016            has ADP max/min Ratio .....        3.8 prolat
PLAT234_ALERT_4_C Large Hirshfeld Difference F015     --C01M     .       0.19 Ang.
PLAT234_ALERT_4_C Large Hirshfeld Difference C00Z     --C01G     .       0.17 Ang.
PLAT241_ALERT_2_C High   'MainMol' Ueq as Compared to Neighbors of       O00J Check
PLAT241_ALERT_2_C High   'MainMol' Ueq as Compared to Neighbors of       O00K Check
PLAT242_ALERT_2_C Low    'MainMol' Ueq as Compared to Neighbors of       Ag01 Check
PLAT342_ALERT_3_C Low Bond Precision on  C-C Bonds ..............    0.01136 Ang.
PLAT351_ALERT_3_C Long   C-H (X0.96,N1.08A)  C01J     - H01G     .       1.11 Ang.
PLAT906_ALERT_3_C Large K Value in the Analysis of Variance ......      2.234 Check
PLAT911_ALERT_3_C Missing FCF Refl Between Thmin & STh/L=    0.600          7 Report
               10  1  3,    6 13 12,   -8 10 16,    5 12 16,    1 17 16,  -2  9 18,
               -7  7 21,
PLAT971_ALERT_2_C Check Calcd Resid. Dens.  0.81Ang From Ag02            1.86 eA-3
PLAT976_ALERT_2_C Check Calcd Resid. Dens.  0.68Ang From O008    .      -0.63 eA-3
```

---

## Alert level G

```
FORMU01_ALERT_1_G  There is a discrepancy between the atom counts in the
            _chemical_formula_sum and _chemical_formula_moiety. This is
            usually due to the moiety formula being in the wrong format.
            Atom count from _chemical_formula_sum:   C27 H15 Ag3 F18 N6 O4
            Atom count from _chemical_formula_moiety:C27 H17 Ag3 F18 N6 O4
FORMU01_ALERT_2_G  There is a discrepancy between the atom counts in the
            _chemical_formula_sum and the formula from the _atom_site* data.
            Atom count from _chemical_formula_sum:C27 H15 Ag3 F18 N6 O4
            Atom count from the _atom_site data:  C27 H17 Ag3 F18 N6 O4
CELLZ01_ALERT_1_G Difference between formula and atom_site contents detected.
CELLZ01_ALERT_1_G ALERT: Large difference may be due to a
            symmetry error - see SYMMG tests
           From the CIF: _cell_formula_units_Z    4
           From the CIF: _chemical_formula_sum  C27 H15 Ag3 F18 N6 O4
```

```
          TEST: Compare cell contents of formula and atom_site data

          atom    Z*formula  cif sites diff
          C        108.00    108.00    0.00
          H         60.00     68.00   -8.00
          Ag        12.00     12.00    0.00
          F         72.00     72.00    0.00
          N         24.00     24.00    0.00
          O         16.00     16.00    0.00
PLAT002_ALERT_2_G Number of Distance or Angle Restraints on AtSite          2 Note
PLAT083_ALERT_2_G SHELXL Second Parameter in WGHT  Unusually Large      12.49 Why ?
PLAT164_ALERT_4_G Nr. of Refined C-H H-Atoms in Heavy-Atom Struct.          2 Note
PLAT172_ALERT_4_G The CIF-Embedded .res File Contains DFIX Records          1 Report
PLAT242_ALERT_2_G Low    'MainMol' Ueq as Compared to Neighbors of       C01A Check
PLAT242_ALERT_2_G Low    'MainMol' Ueq as Compared to Neighbors of       C01C Check
PLAT242_ALERT_2_G Low    'MainMol' Ueq as Compared to Neighbors of       C01E Check
PLAT242_ALERT_2_G Low    'MainMol' Ueq as Compared to Neighbors of       C01H Check
PLAT242_ALERT_2_G Low    'MainMol' Ueq as Compared to Neighbors of       C01K Check
PLAT242_ALERT_2_G Low    'MainMol' Ueq as Compared to Neighbors of       C01M Check
PLAT434_ALERT_2_G Short Inter HL..HL Contact F00N     ..F019     .       2.83 Ang.
                                               -x,1-y,1-z  =      3_566 Check
PLAT720_ALERT_4_G Number of Unusual/Non-Standard Labels ..........         75 Note
             Ag01    Ag02    Ag03    F004    N005    N006    F007    O008
             N009    N00A    N00B    N00C    F00D    F00E    O00F    F00G
             F00H    F00I    O00J    O00K    C00L    H00L    F00M    F00N
             F00O    C00P    H00P    F00Q    F00R    F00S    C00T    C00U
             C00V    C00W    C00X    H00A    H00B    H00C    C00Y    C00Z
             F010    C011    C012    C013    C014    F015    F016    C017
             H017    C018    H018    F019    C01A    C01B    C01C    F01D
             C01E    C01F    H01A    H01B    H01C    C01G    H01D    H01E
             C01H    C01I    C01J    C01K    C01L    H01H    H01I    H01J
             C01M    H01F    H01G
PLAT860_ALERT_3_G Number of Least-Squares Restraints ............           1 Note
PLAT883_ALERT_1_G Absent Datum for _atom_sites_solution_primary ..     Please Do !
PLAT912_ALERT_4_G Missing # of FCF Reflections Above STh/L=  0.600        290 Note
PLAT941_ALERT_3_G Average HKL Measurement Multiplicity ..........         3.1 Low
PLAT969_ALERT_5_G The 'Henn et al.' R-Factor-gap value ..........       4.434 Note
             Predicted wR2: Based on SigI**2  3.40 or SHELX Weight 14.55
PLAT978_ALERT_2_G Number C-C Bonds with Positive Residual Density.          0 Info
```

---

0 **ALERT level A** = Most likely a serious problem - resolve or explain
0 **ALERT level B** = A potentially serious problem, consider carefully
19 **ALERT level C** = Check. Ensure it is not caused by an omission or oversight
22 **ALERT level G** = General information/check it is not something unexpected

8 ALERT type 1 CIF construction/syntax error, inconsistent or missing data
20 ALERT type 2 Indicator that the structure model may be wrong or deficient
6 ALERT type 3 Indicator that the structure quality may be low
6 ALERT type 4 Improvement, methodology, query or suggestion
1 ALERT type 5 Informative message, check

---

## Validation response form

Please find below a validation response form (VRF) that can be filled in and pasted into your CIF.

```
# start Validation Reply Form
_vrf_PLAT041_lcz032
;
PROBLEM: Calc. and Reported SumFormula    Strings  Differ     Please Check
RESPONSE: ...
;
_vrf_PLAT042_lcz032
;
PROBLEM: Calc. and Reported MoietyFormula Strings  Differ     Please Check
RESPONSE: ...
;
_vrf_PLAT043_lcz032
;
PROBLEM: Calculated and Reported Mol. Weight Differ by ..       2.02 Check
RESPONSE: ...
;
_vrf_PLAT068_lcz032
;
PROBLEM: Reported F000 Differs from Calcd (or Missing)...     Please Check
RESPONSE: ...
;
_vrf_PLAT094_lcz032
;
PROBLEM: Ratio of Maximum / Minimum Residual Density ....       2.07 Report
RESPONSE: ...
;
_vrf_PLAT213_lcz032
;
PROBLEM: Atom F010            has ADP max/min Ratio .....        3.2 prolat
RESPONSE: ...
;
_vrf_PLAT234_lcz032
;
PROBLEM: Large Hirshfeld Difference F015     --C01M     .       0.19 Ang.
RESPONSE: ...
;
_vrf_PLAT241_lcz032
;
PROBLEM: High   'MainMol' Ueq as Compared to Neighbors of       O00J Check
RESPONSE: ...
;
_vrf_PLAT242_lcz032
;
PROBLEM: Low    'MainMol' Ueq as Compared to Neighbors of       Ag01 Check
RESPONSE: ...
;
_vrf_PLAT342_lcz032
;
PROBLEM: Low Bond Precision on  C-C Bonds ..............    0.01136 Ang.
RESPONSE: ...
;
_vrf_PLAT351_lcz032
;
PROBLEM: Long   C-H (X0.96,N1.08A)  C01J     - H01G     .       1.11 Ang.
RESPONSE: ...
```

```
;
_vrf_PLAT906_lcz032
;
PROBLEM: Large K Value in the Analysis of Variance ......      2.234 Check
RESPONSE: ...
;
_vrf_PLAT911_lcz032
;
PROBLEM: Missing FCF Refl Between Thmin & STh/L=    0.600           7 Report
RESPONSE: ...
;
_vrf_PLAT971_lcz032
;
PROBLEM: Check Calcd Resid. Dens.  0.81Ang From Ag02            1.86 eA-3
RESPONSE: ...
;
_vrf_PLAT976_lcz032
;
PROBLEM: Check Calcd Resid. Dens.  0.68Ang From O008    .      -0.63 eA-3
RESPONSE: ...
;
# end Validation Reply Form
```

---

It is advisable to attempt to resolve as many as possible of the alerts in all categories. Often the minor alerts point to easily fixed oversights, errors and omissions in your CIF or refinement strategy, so attention to these fine details can be worthwhile. It is up to the individual to critically assess their own results and, if necessary, seek expert advice.

---

**PLATON version of 17/09/2025; check.def file version of 17/09/2025**

---

# duplicate check

**No duplication found**

---

**Datablock lcz032** - ellipsoid plot

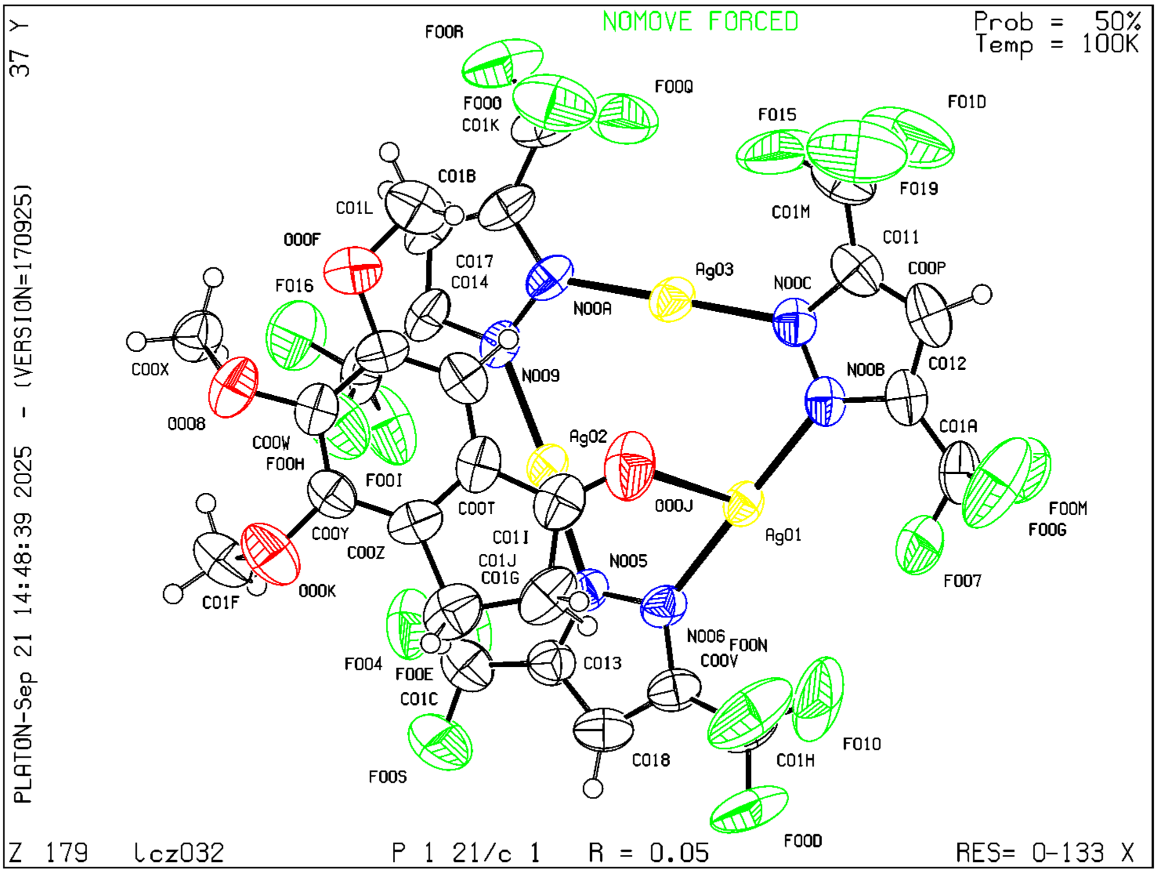

Supplement: Supplementary file 2 — Supporting File 2: anie72462‐sup‐0002‐DataFile.zip. [file ANIE-65-e1218503-s002.zip › supplementary_materials_crystallographic_data submit.zip!/cif_and_checkcif(1-114)/Ag3Pz3_32/Ag3Pz3_32_checkcif.pdf]
